# Nanoconjugates of a calixresorcinarene derivative with methoxy poly(ethylene glycol) fragments for drug encapsulation

**DOI:** 10.3762/bjnano.9.195

**Published:** 2018-07-27

**Authors:** Alina M Ermakova, Julia E Morozova, Yana V Shalaeva, Victor V Syakaev, Aidar T Gubaidullin, Alexandra D Voloshina, Vladimir V Zobov, Irek R Nizameev, Olga B Bazanova, Igor S Antipin, Alexander I Konovalov

**Affiliations:** 1 Arbuzov Institute of Organic and Physical Chemistry, FRC Kazan Scientific Center of RAS, Arbuzov str. 8, 420088 Kazan, Russian Federation; 2 Kazan Federal University, Kremlevskaya st. 18, 420008 Kazan, Russian Federation; 3 Kazan National Research Technical University named after A. N. Tupolev – KAI, K. Marx str. 10, 420111 Kazan, Russian Federation

**Keywords:** calixresorcinarene, drug encapsulation, hemotoxicity, methoxy poly(ethylene glycol), temperature-controlled release

## Abstract

In order to obtain a non-toxic amphiphilic calixresorcinarene capable to form nanoconjugates for drug encapsulation, tetraundecylcalixresorcinarene functionalized by methoxy poly(ethylene glycol) chains has been synthesized. The macrocycle obtained is characterized by low hemotoxicity. In aqueous solution it forms nanoassociates that are able to encapsulate organic substrates of different hydrophobicity, including drugs (doxorubicin, naproxen, ibuprofen, quercetin). The micelles of the macrocycle slowed down the release of the hydrophilic substrates in vitro. In physiological sodium chloride solution and phosphate-buffered saline, the micelles of the macrocycle acquire thermoresponsive properties and exhibit a temperature-controlled release of doxorubicin in vitro. The combination of the low toxicity and the encapsulation properties of the obtained calixresorcinarene–mPEG conjugate shows promising potential for the use as a supramolecular drug-delivery system.

## Introduction

One of the acute problems of modern medicinal therapy is the development of novel drug-delivery systems with low toxicity that have sizes of ≤100 nm, increase biocompatibility, stability and circulation time of the drugs, and are sensitive to release stimuli. Nanocontainers on the base of amphiphilic compounds combine these properties and are able to increase the solubility of hydrophobic drugs due to their binding near the hydrophobic core, whereas the shell, which is composed of hydrophilic groups, ensures the stability of the system in solution. Examples for these amphiphilic compounds are surfactants [1], liposomes [2], polymers [3], and synthetic macrocycles [4]. Calixarenes and calixresorcinarenes are outstanding class of synthetic macrocycles. With regard to supramolecular containers for drug encapsulation they offer a convenient platform that allows for the functionalization of upper and lower rim by different groups, offers an aromatic cavity capable to π–π, CH–π and cation–π interactions and can form exo- and endo-complexes with substrates [5]. Moreover, the conformation can be controlled through influencing the parameters for the self-organisation of amphiphiles.

In general, the presence of long alkyl groups on the lower rim of the calixresorcinarene platform yields boat or cone conformations of the macrocycle. This, in turn, leads to a micelle-like self-association of the macrocycles, the upper rim of which are functionalized by hydrophilic substituents. Thus, the self-organised structures of amphiphilic calixresorcinarenes can effectively encapsulate organic substrates trough the formation of co-associates [6–9], demonstrating their ability to form supramolecular nanocontainers. The toxicity of such supramolecular nanocontainers remains a relevant challenge.

One of the approaches for the introduction of hydrophilic groups into the macrocycle and to decrease its toxicity is the usage of fragments of known biocompatible polymers, such as PEG, polylactic acid and polycaprolactone. The functionalization of the calixresorcinarene platform with such fragments leads to the formation of hyperbranched three-dimensional structures, which exhibit a higher solubility, lower viscosity and higher population of ending groups than linear polymers. Earlier, syntheses of amphiphilic derivatives of calixarenes and oligolactic acid [10–11], calixarenes and PEG [12–18], calixarene and calixresorcinarene polylactides [19] and polycaprolactones [20–21], as well as block copolymers on the base of calixresorcinarene [22] were described. However, the investigations of calixresorcinarene–polymer conjugates involving synthesis, the study of toxicity and the encapsulation of guests are very limited [22].

It is known that PEG is the most commonly used polymer in the production of various therapeutics and nanomaterials due to a number of benefits among which its low toxicity the most important [23–24]. PEG has good solubility in water and organic solvents. When being a component of polymeric micelles, it forms the outer hydrophilic shell of the micelle, contributes the colloidal stability of the nanocontainer and prevents premature release and biodegradation of the drug; the conjugation of PEG and calixresorcinarene will yield an amphiphile with improved binding properties due to the contribution of macrocycle platform to the interaction with substrates. This can lead to an increase of the release time of the drug bound by calixresorcinarene–PEG conjugate micelles. Thus, the aim of our investigation was synthesis of amphiphilic calixresorcinarene, functionalized by PEG fragments and the study of its potential as a low-toxicity supramolecular nanocontainer for drugs.

## Results and Discussion

### Synthesis and characterization of tetraundecylcalix[4]resorcinarene–mPEG conjugate

**Scheme 1:**
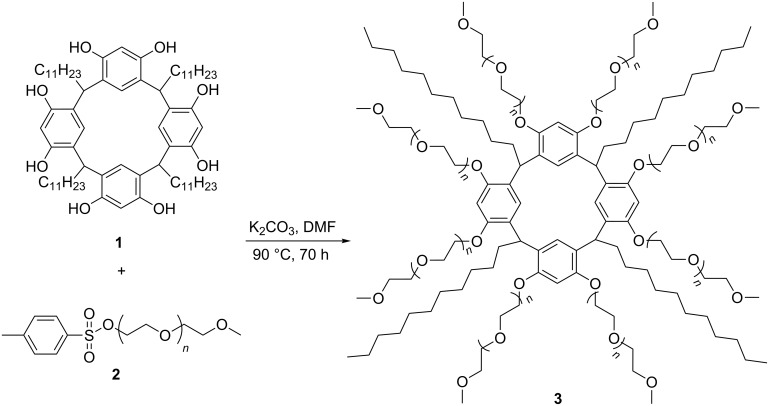
Synthesis of tetraundecylcalix[4]resorcinarene–mPEG conjugate **3**.

**Figure 1:**
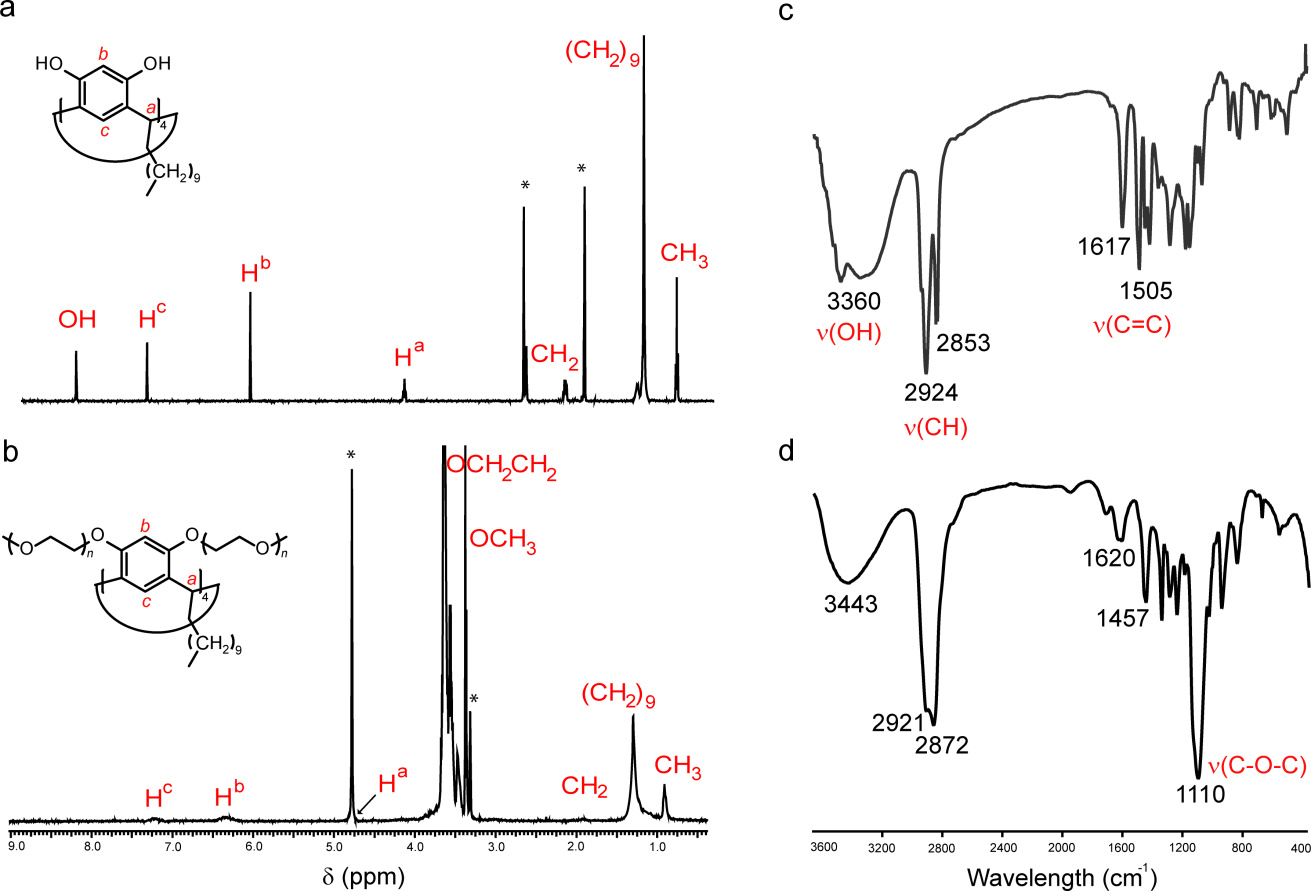
(a, b) ^1^H NMR spectra of (a) macrocycle **1** in acetone-*d*_6_ and (b) macrocycle **3** in CD_3_OD; (c, d) FTIR spectra of (c) **1** and (d) **3**.

To obtain the amphiphilic calixresorcinarene, tetraundecylcalix[4]resorcinarene **1** was functionalized with tosylated mPEG-550 (**2**) (Scheme 1). The obtained macrocycle **3** was characterized by ^1^H NMR, ^13^С NMR and FTIR. Its molecular weight was measured by static light scattering (SLS) and MALDI-TOF. In the ^1^H NMR spectrum of **3** signals of ethyleneoxy and methoxy groups at 3.65 and 3.37 ppm, respectively, are observed; signals of bridged methylidene groups and aromatic groups are very broad (Figure 1b). Also a strong broadening of signals of aromatic groups is observed in ^13^С NMR spectra (Figure S1, Supporting Information File 1). Thus, the attachment of polymeric groups to the calixresorcinarene platform leads to a significant slowdown of the rate of the conformational boat–cone–boat interconversion in the NMR time scale and, consequently, to the broadening of the signals of calixresorcinarene platform in the NMR spectra. The same broadening in the spectra of tetraundecylcalix[4]resorcinarene with poly-ε-caprolactone fragments on the upper rim was described earlier [22].

In the FTIR spectrum of **3**, the strong band of stretching vibrations of the C–O–C groups of PEG at 1110 cm^−1^ appears, confirming the conjugation of macrocycle and PEG (Figure 1d). In the ^1^H NMR spectrum of **3**, the ratios between the integral intensities of terminal methoxy groups (at 3.37 ppm) and methyl and (CH_2_)_9_ groups (at 0.91 and 1.30 ppm, respectively) testify the complete substitution of the hydroxyl groups of macrocycle **1**. They also allows for the estimation of the quantity of attached ethyleneoxy groups and of an average molecular mass of **3** (Figure S2, Supporting Information File 1). The integral intensity of the signal at 3.65 ppm corresponds to approximately 86 ethyleneoxy groups per one macrocycle molecule, which gives an average molecular mass of about 5.0 kDa. The determination of the molecular weight by using SLS method gives a value of 4.7 ± 0.7 kDa (Figure S3, Supporting Information File 1). MALDI-TOF mass spectra display molecular weight distributions from 2.6 to 4.2 kDa (Figure S4, Supporting Information File 1). The molecular weight value determined by SLS correlates with ^1^H NMR data but is higher than the value obtained by mass spectroscopy. The same tendency was described by Corbin et al for calixarene and calixresorcinarene polylactide star polymers [19].

### Self-association and hemotoxicity of **3**

**Figure 2:**
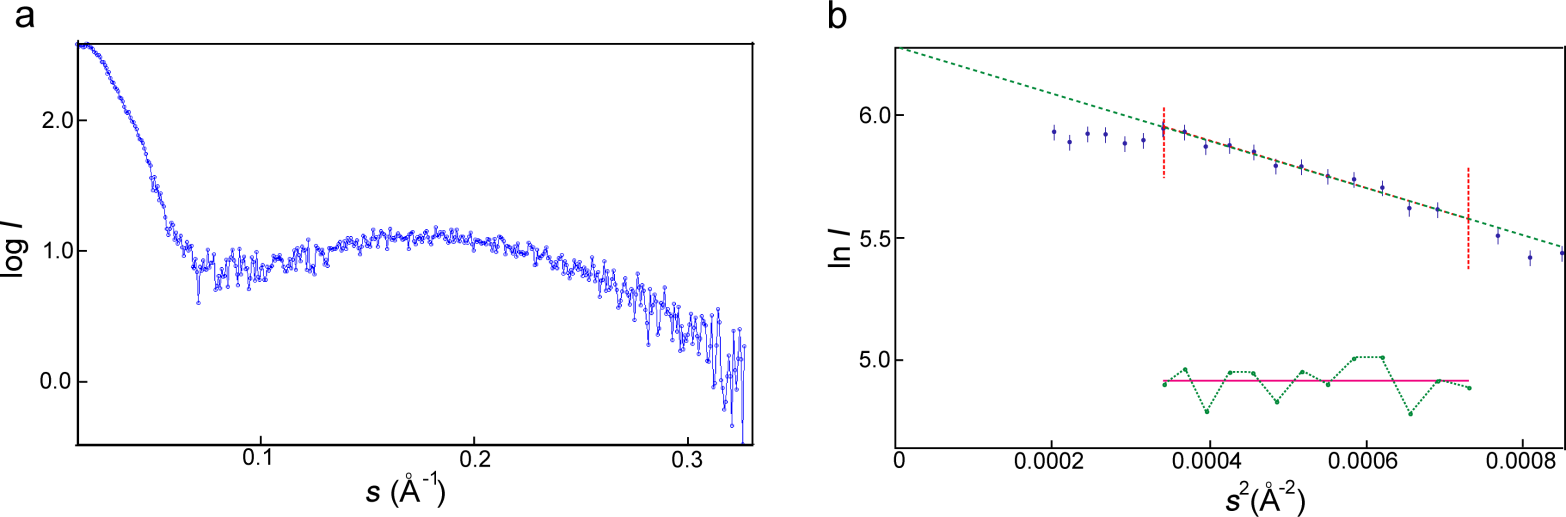
(a) SAXS diffraction intensity profile of an aqueous solution of **3** (*c***_3_** = 137 mg/mL) at 23 °C (logarithmic scale) after subtraction of the background scattering (water); scattering vector *s* = 4π·sinθ/λ (Å^−1^); X-ray wavelength λ = 1.5418 Å, (b) Guinier plot ln(*I*) as a function of *s*^2^ with linear regression of the curve; *R*_g_ = 53.5 ± 1.8 Å, *I*(0) = 531 ± 18, *r* = 0.98.

First, the self-association of macrocycle **3** in the aqueous solution was studied by using small-angle X-ray scattering (SAXS). The obtained data are typical for core–shell particles that practically do not interact with each other (Figure S5, Supporting Information File 1). Figure 2a presents the resulting scattering curve of the aqueous solution of **3** after the subtraction of water scattering. The character of scattering corresponds to a monodisperse particle system. Power-law slopes from the data in the high-*s* region can be used to describe the morphology of such a particle system. One of the important features deduced from the power-law slopes is the radius of gyration (*R*_g_) [25], which is the value of the square root of the average squared distances of each scatterer from the particle centre. The radius of gyration *R*_g_ and the forward scattering *I*(0) were calculated by using the Guinier plot (Figure 2b) and are 53.5 ± 1.8 Å and 531 ± 18, respectively.

Additionally, with the help of the PRIMUS software [26] the fitting of SAXS spectra in a globular model with a local monodisperse approximation was performed and the distance distribution function (*P*(*r*)) of the particles was calculated (Figure S6, Supporting Information File 1). The SAXS intensity distribution (Figure S6a, Supporting Information File 1) and the corresponding distance distribution function (Figure S6b, Supporting Information File 1) are typical for the structure with a core of smaller electron density than the solvent and with shell of a larger electron density than the solvent (the known values of electron densities for water, CH_2_ chains and PEG are 0.33 e/Å^3^, 0.274 e/Å^3^ and 0.4 e/Å^3^ [27], respectively). The fitting of the data obtained using the GNOM software [28] gave the following parameters: reciprocal space radius of gyration *R*_g_ = 55.58 Å, *I*(0) = 539.2, real space *R*_g_ = 55.41 ± 0.26 Å, *I*(0) = 546.7 ± 5.4, maximum characteristic size of the particles (*D*_max_) = 150 Å (Figure S6, Supporting Information File 1). The values of *R*_g_, obtained by two methods, are quite close to the value calculated from the Guinier plot and, in the case of a sphere-shape model, correspond to an average effective particles radius of 71.5 Å (diameter about 143 Å). The volume occupied by the hydrated particles in the solution were computed using the Porod invariant asymptotics and is about 82301 Å^3^. The theoretic volume of macrocycle **3** (with a maximum of 12 oxyethylene groups in the mPEG chains) is about 5556.2 and 5298.0 Å^3^ calculated by two methods [29–30] using the Molecular Modelling Pro software. Therefore, 14–15 molecules of macrocycle **3** can participate in the formation of core–shell particles in aqueous solution.

The critical association concentration (cac) value of **3** was obtained by using the fluorescent pyrene-probe method. It amounts to 0.01 mg/mL (Figure S8, Supporting Information File 1). mPEG-550 does not form self-organised structures because of its high hydrophilicity and the lack of hydrophobic groups.

The study of the hemolytic activity of **3** against human red blood cells (hRBC) demonstrates the low toxicity of its micelles in a concentration range above the cac value of **3** (Table 1).

**Table 1:** Hemolytic activity data of **3** against human red blood cells (hRBC) in 0.15 M NaCl.

*c*, mg/mL	hemolysis of hRBC, %

5.4	0.5
2.7	0
1.4	0
control (0.15 M NaCl)	0
H_2_O	100

**Figure 3:**
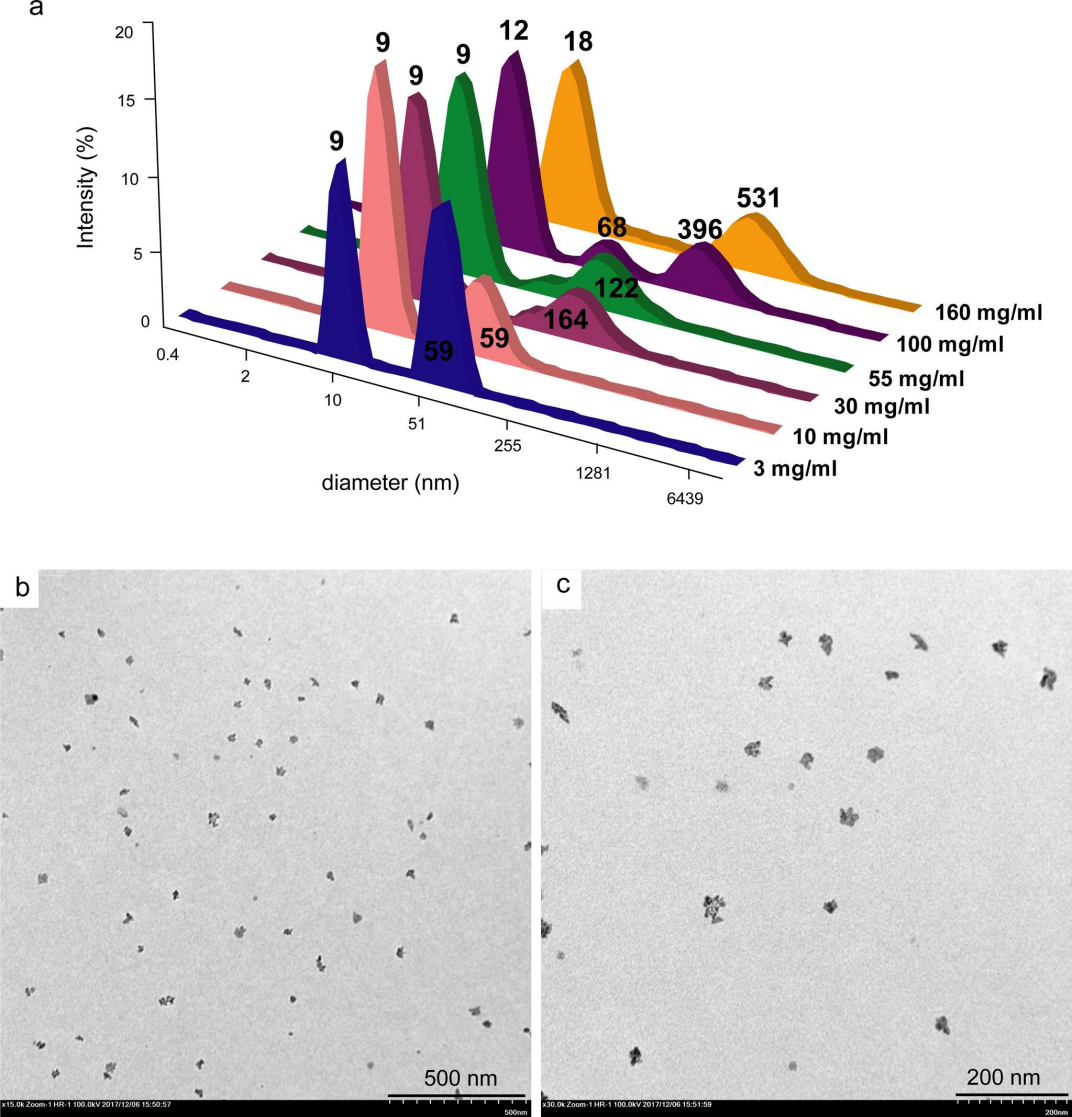
(a) The intensity-averaged particle size distribution of aqueous solutions of **3** at different concentrations; (b, c) TEM images of particles of **3**, scale bars: (b) 500 nm, (c) 200 nm, *c***_3_** = 10 mg/mL).

The self-association of **3** was further studied by using dynamic light scattering (DLS) and a the bimodal particle size distribution of **3** was observed in aqueous solution. The detailed DLS data are presented in Table S1, Supporting Information File 1. In Figure 3a the intensity-averaged size distribution is presented. It shows that in the solution are mainly small particles with average diameters from 9 to 18 nm, depending on the macrocycle concentration (3–160 mg/mL). Obviously, these are micellar-like self-organised structures of macrocycle **3** with a hydrophobic core formed by alkyl groups and a hydrophilic shell formed by mPEG chains. It is possible that big particles (with average diameters from 59 to 531 nm) are co-associates of micelles of **3**, so-called “multimicelle aggregates” [31], which emerge as a result of interactions between the ethyleneoxy groups. TEM images show the presence of particles with diameters about 5–10 nm (Figure 3b,c), showing the predominance of small particles from the self-association of **3**. The ξ-potential value of **3** is −10.1 mV (Figure S9, Supporting Information File 1). This probably indicates the partial ionization of ethyleneoxy groups due residual potassium cations, which easily form a complex with mPEG fragments [32] and appear due to the reaction conditions (see Experimental section).

Because of the presence of mPEG groups, compound **3** can be considered a non-ionic surfactant. It is known, that in aqueous solutions of non-ionic surfactants increasing temperatures leads to phase separation. There are two phases at the critical temperature (cloud point, *T*_c_) in the solution, one of which is saturated by surfactant molecules and another one is almost surfactant-free. At *T*_c_, dehydration of hydrophilic groups occurs and the interaction of inter- and intramolecular H-bonds among the surfactant is increased, which leads to further aggregation. The addition of inorganic and organic compounds influences the value of *Т*_с_ [33].

**Figure 4:**
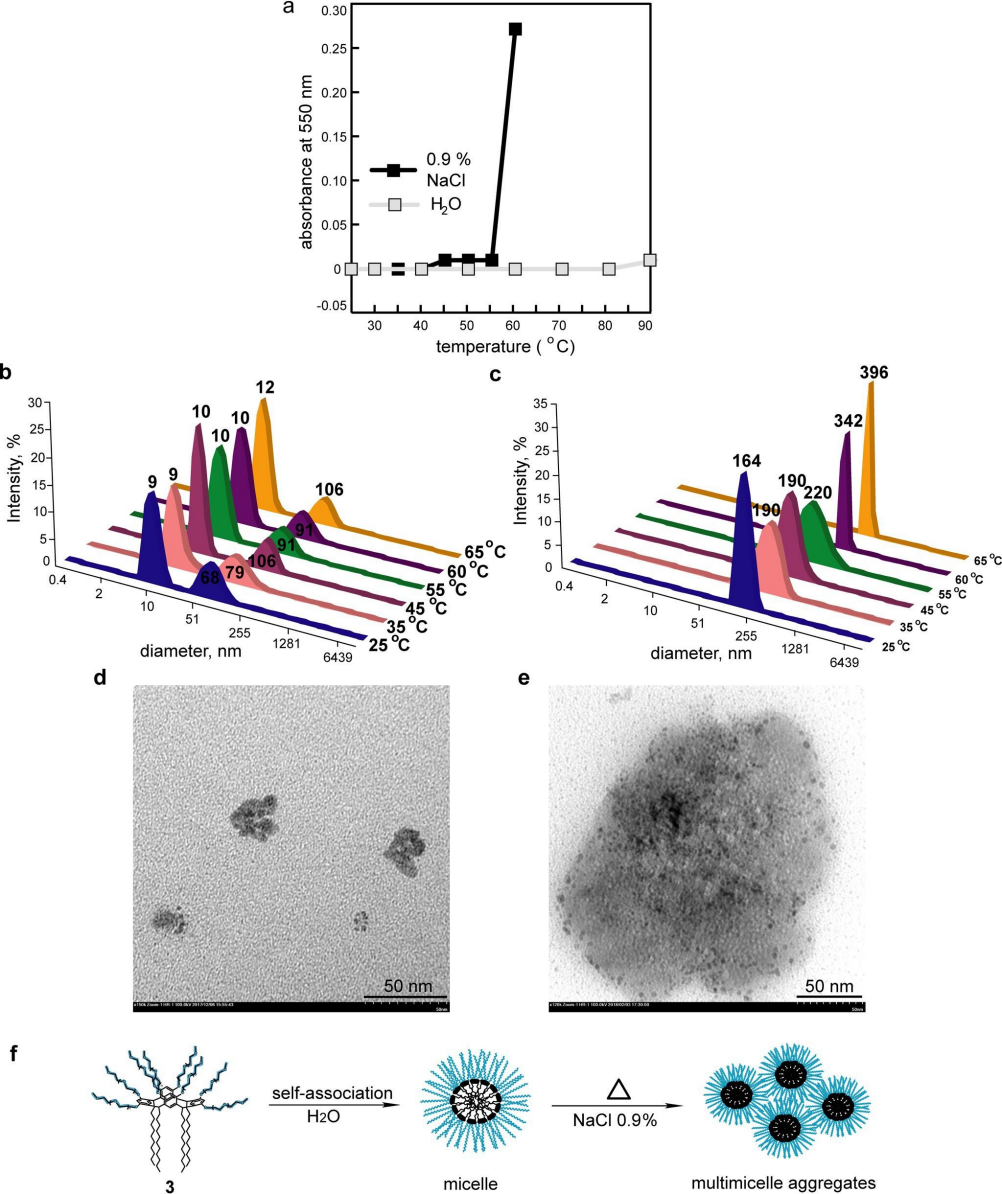
(a) The dependence of the optical density of aqueous solutions of **3** at 550 nm on the solution temperature in the absence and the presence of 0.9% NaCl; (b, c) intensity-averaged particle size distribution of aqueous solutions of **3** in (b) the absence and (c) in the presence of 0.9% NaCl; (d, e) TEM images of **3** particles in the (d) absence and (e) the presence of 0.9% NaCl; scale bars: 50 nm, *c***_3_** = 10 mg/mL); (f) proposed scheme of self-association of macrocycle **3**.

The influence of the temperature on the phase behavior of an aqueous solution of **3** (10 mg/mL) was performed. Heating of the aqueous solution of **3** in the absence of any salts did not cause a phase separation up to 90 °C, while solutions of **3** in a physiological solution of sodium chloride (0.9% NaCl) solution or in phosphate-buffered saline (PBS, pH 7.4) clouded at 60 °C (Figure 4a). DLS method showed that an increase of the particle size of **3** in the presence of salts occurred already at room temperature (Figure 4c, Figure S10, Supporting Information File 1). In the 0.9% NaCl solution a monomodal particle size distribution was observed (Table S1, Supporting Information File 1). TEM images of dried saline solution of **3** confirmed the growing of the macrocycle particle size to about 200 nm (Figure 4e). Obviously, in the presence of inorganic salts the aggregation of micelles of **3** into “multimicelle aggregates” [31] occurred due to the amplification of the hydrophobic effect and the increase of the role of inter- and intramolecular interactions. Heating of the solutions leads to a further increase of the particles size. Thus, in aqueous solution macrocycle **3** forms micelles with a diameter of 5–10 nm, which associate into “multimicelle aggregates” in the presence of salts (0.9% NaCl, PBS) and become sensitive to a change of the solution temperature (Figure 4f).

### Micelles of **3** in the encapsulation of hydrophobic substrates

The encapsulation of model compounds of different hydrophobicity by macrocycle **3** was studied (Table 2). These compounds were doxorubicin (Dox), an anthracycline antitumor antibiotic, quercetin (QC), a flavonol that exhibits decongestant, antispasmodic, antihistaminic, anti-inflammatory and potential antitumor effects, the nonsteroidal anti-inflammatory drugs ibuprofen (IF) and naproxen (Nap), and Orange OT, a hydrophobic optical probe. The compounds have the ability for non-covalent interactions with **3** through π–π and СH–π interactions with the aromatic cavity of macrocycle, the hydrophobic effect with its alkyl substituents and hydrogen, and donor–acceptor bonds with its polyethyleneoxy groups.

**Table 2:** Data of substrate encapsulation in micelles of **3**; log *P*^a^: octanol–water partition coefficient of the substrates; *m***^3^**: mass of **3** (mg); *m*^S0^: initial mass of the substrate (mg); *m*^S1^: mass of solubilized substrate (mg); *EE*: encapsulation efﬁciency value (%)^b^; *DL*: drug-loading value (%)^b^.

	log *P*	*m***^3^** (mg)	*m*^S0^ (mg)	*m*^S1^ (mg)	*EE* (%)	*DL* (%)

Orange OT^c^	5.19	10	1	0.176	17.6	1.7
		6.3	3	0.391	13.0	5.8
QC^c^	1.81	10	1	0.06	6.0	0.6
		3.5	1	0.05	5.0	1.4
		1	1	0.045	4.5	4.3
Dox^c^	1.41	30	5	1.3	26.0	4.15
		30	1	0.44	44.0	1.45
		3.8	1.6	0.3	18.75	7.3
IF^d^	3.50	10	10	1.9	19	16
Nap^d^	3.29	10	10	7.5	75	42.9
RhB^c^	—	150	30	2.5	8.3	1.6

^a^ log *P* values calculated using the ALOGPS 2.1 software [34]; ^b^ *EE* and *DL* values are calculated as described in the Experimental section; ^c^thin-film hydration; ^d^solubilization.

The encapsulation was performed through thin-film hydration (also called evaporation method) or through solubilisation. Thin-film hydration means that ethanol solutions of macrocycle and substrate were mixed and dried. The obtained film was redissolved in bidistilled water, and the non-soluble substrate was removed. Otherwise the substrates were solubilized in aqueous (or D_2_O) solutions of **3**. The encapsulation data are collected in Table 2. To highlight the effect of the macrocycle platform on substrate binding, **3** and mPEG-550 were compared regarding their influence on physico-chemical properties of the substrates by using UV–vis spectroscopy and fluorimetry). The influence of substrate encapsulation on the size of micelles of **3** was determined by using DLS (Table S1, Supporting Information File 1).

**Figure 5:**
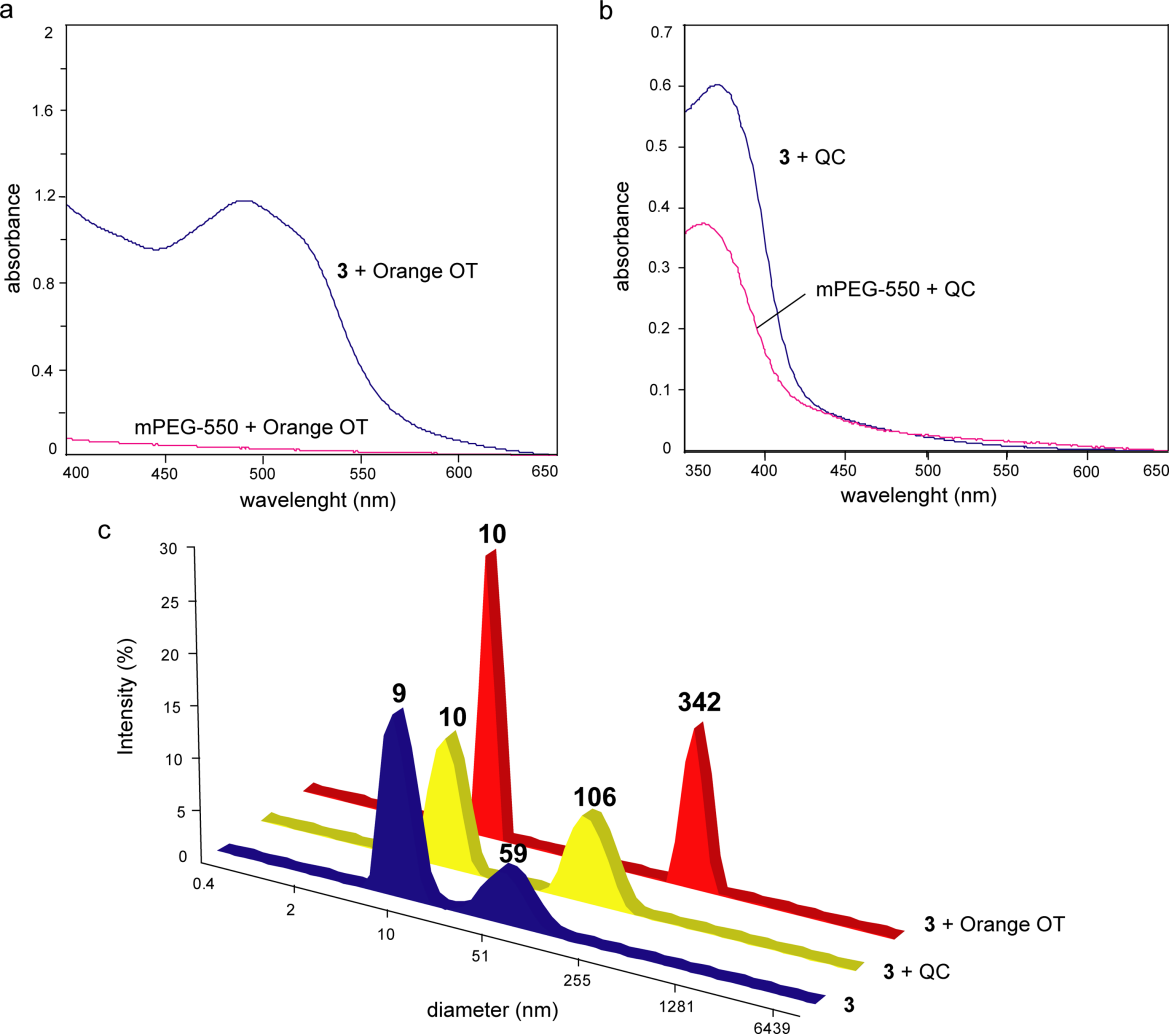
(a, b) Absorption spectra of Orange OT (a) and QC (b) in aqueous solutions of **3** and mPEG-550 (solubilisation method, *c***_3_** = 10 mg/mL, *c*_mPEG-550_ = 22 mg/mL, quartz cells with an optical path of 0.2 cm); (c) intensity-averaged particle size distribution of aqueous solutions of **3** in the absence and the presence of Orange OT and QC (thin-film hydration method, *c***_3_** = 10 mg/mL).

The solubilisation of Orange OT and QC in aqueous solutions of **3** and mPEG-550 demonstrated the advantage of the macrocycle in the encapsulation of these substrates (Figure 5a,b). UV–vis spectra of the obtained solutions showed a significant increase of the solubility of hydrophobic Orange OT in the presence of **3** as a result of dye encapsulation into the hydrophobic core of macrocyle micelles. In the case of the more hydrophilic QC, a moderate increase of its absorbance in the solution of **3** is observed. But the bathochromic shift of the QC absorption maximum in the solution of **3** (370 nm) compared to the mPEG-550 solution (362 nm) means a more hydrophobic microenvironment of the substrate in the macrocycle micelles [35]. The encapsulation of these substrates through thin-film hydration showed that the encapsulation efficiency (*EE*) of more hydrophobic Orange OT is a higher than that of QC, probably, due to a higher contribution of the hydrophobic effects to the binding of Orange OT (Table 2). The *EE* value of QC grows after increasing the initial mass of the macrocycle (*m***^3^**). According to the DLS data, during encapsulation of these substrates, an increase of the particle size of **3** is observed, which is probably related to an increased hydrophobicity of the particles. The higher drug-loading (*DL*) values of Orange OT lead to a stronger growth of the particles (Figure 5c, Table S1, Supporting Information File 1).

The encapsulation of Dox into micelles of **3** was also carried out by using thin-film hydration. The particle size distribution of **3** significantly changes in the presence of Dox: The averaged size of small particles increases from 8 to 16 nm and large particles disappear, which leads to a decrease of the PDI value (Figure S11a, Table S1, Supporting Information File 1). By using fluorescence it was shown that in contrast to the mPEG-550 solution, the binding of Dox in the solution of **3** leads to the quenching of its fluorescence (Figure S11b–d, Supporting Information File 1). According to the data presented in Table 2, Dox is solubilized more effectively than QC. Dox is an anthraquinone compound with amino and hydroxy groups, which can form multiple H-bonds with oxygen atoms of the ethyleneoxy groups of the macrocycle. Hence, Dox has more possibilities to interact with micelles of **3**. With the same mass of **3**, the decrease of the initial mass of Dox (*m*^S0^) leads to an increase of the *EE* value and to a decrease of the *DL* value (Table 2). Thus, the increase of amount of macrocycle **3** yields a higher solubility of the substrates.

**Figure 6:**
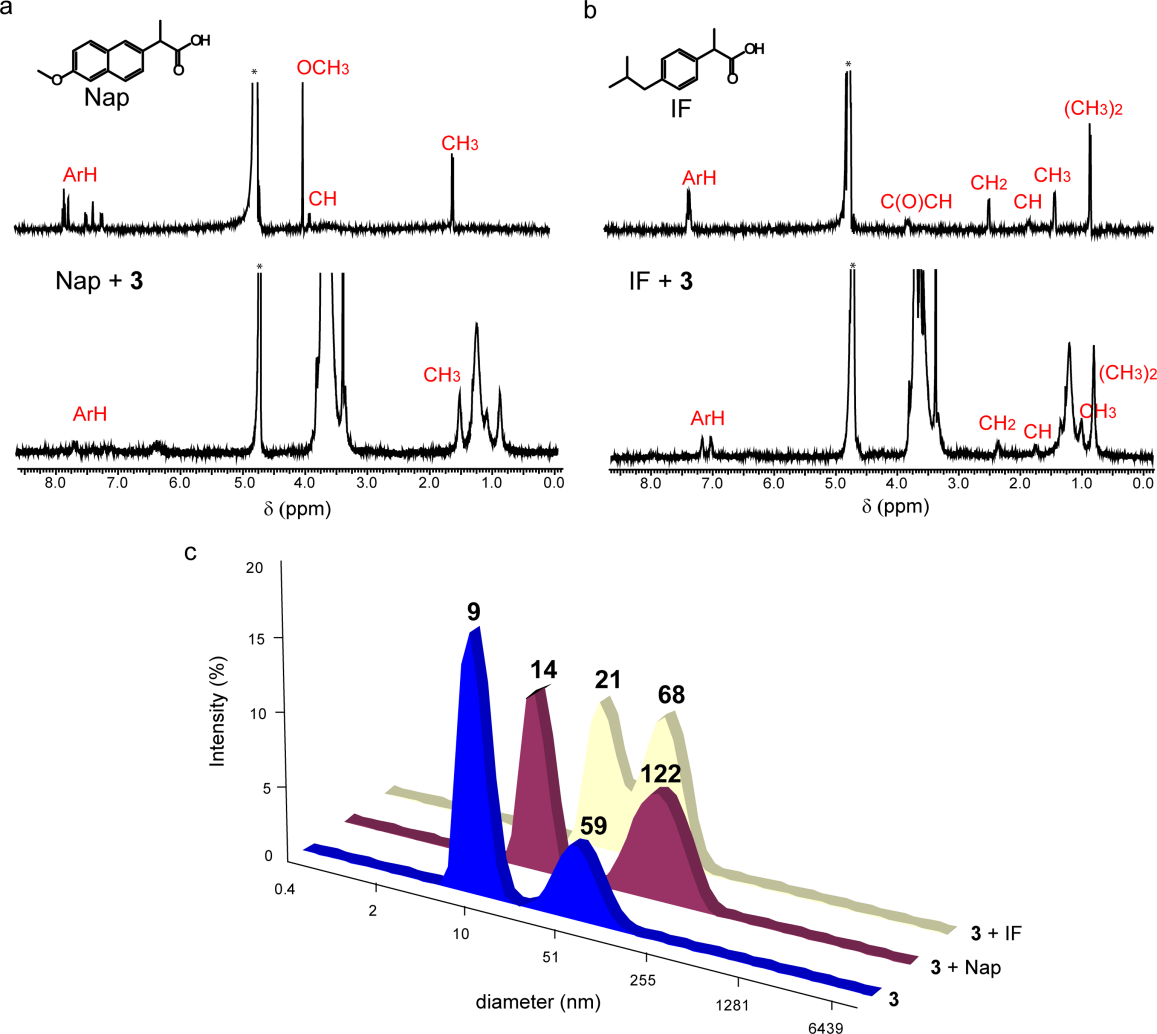
(a) ^1^H NMR spectra of Nap and **3** + Nap solutions in D_2_O; (b) ^1^H NMR spectra of IF and **3** + IF solutions in D_2_O; (c) intensity-averaged particle size distribution of aqueous solutions of **3** in the absence and the presence of Nap and IF (solubilization method, *c***_3_** = 10 mg/mL).

The encapsulation of ibuprofen (IF) and naproxen (Nap) was carried by dissolving them in solutions of **3** in D_2_O at room temperature. The fluorescence emission of naproxen sodium is quenched in the presence of macrocycle and does not change in the presence of mPEG-550 (Figure S12, Supporting Information File 1). This is evidence for the binding of Nap near the aromatic fragments of macrocycle. After the solubilization of IF and Nap, shift of proton signals of the substrates to higher fields in ^1^Н NMR spectra (to −0.13 ppm) as well as line broadening occur. This testifies the binding of the both substrates near the aromatic platform of the macrocycle (Figure 6a,b). DLS shows the growth of the averaged hydrodynamic diameter of the particles (Figure 6c). According to our results, the *EE* and *DL* values of Nap are higher than those of IF. Nap is more hydrophilic than IF and has methyloxy group yielding additional donor–acceptor interactions with the hydrophilic groups of the macrocycle. Moreover, the *EE* and *DL* values of Nap are higher than those of Dox, which can be explained by a lower permeability of the micelles of **3** for the more bulky Dox molecule.

In conclusion, the incorporation of hydrophobic substrates into the micelles of **3** depends on the substrate structure and includes the interaction with both hydrophobic and hydrophilic fragments of macrocycle molecules as well as with the aromatic platform of the macrocycle.

### In vitro release of substrates from micelles of **3**

The hydrophilic dye rhodamin B (RhB) was used as an optical probe for the investigation of the release from micelles of **3**. The quenching of RhB emission in the presence of **3** and the absence of any change in the RhB emission in the presence of mPEG-550 show that a binding of the dye near the aromatic platform of the macrocycle occurred (Figure S13, Supporting Information File 1). The **3** + RhB micelles were prepared by using thin-film hydration; the excess of RhB was removed by dialysis because of the hydrophilic character of dye. The *DL* and *EE* values of RhB in **3** + RhB micelles are presented in Table 2.

**Figure 7:**
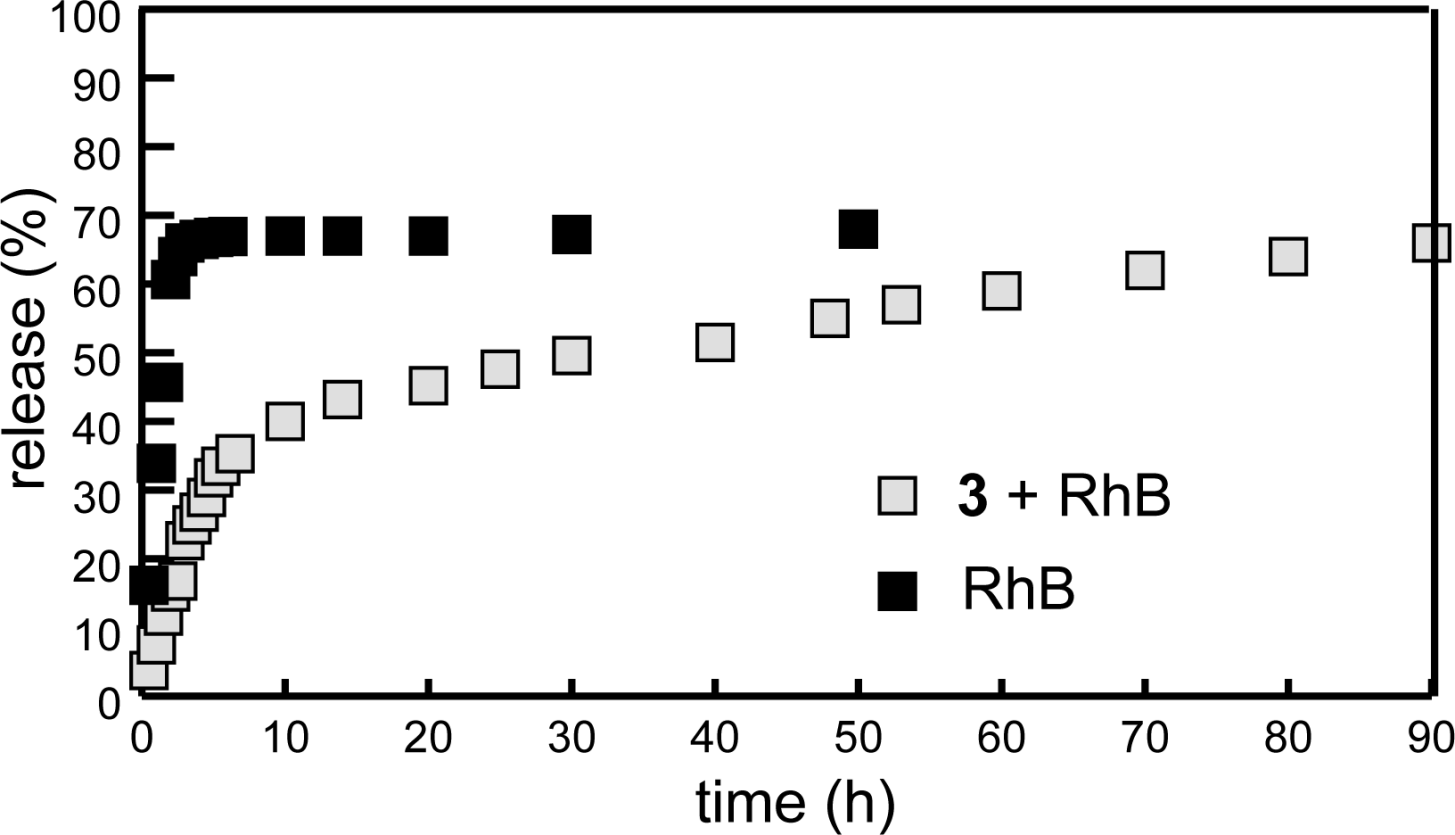
In vitro release proﬁles of free RhB and RhB-loaded micelles of **3** in PBS release medium at pH 7.4.

Figure 7 presents the release proﬁles of RhB from pure solution and from **3** + RhB micellar solution into PBS release medium (pH 7.4). As expected, the **3** + RhB micelles exhibited a slower release rate. In the first 10 h, the released amount of **3** + RhB micelles was around 40%, then the release slowed down and about 30% of the substrate was released over a period of the following 80 h. The slower release of RhB-loaded micelles is attributed to the multimodal binding of the dye in micelles of macrocycle **3**.

**Figure 8:**
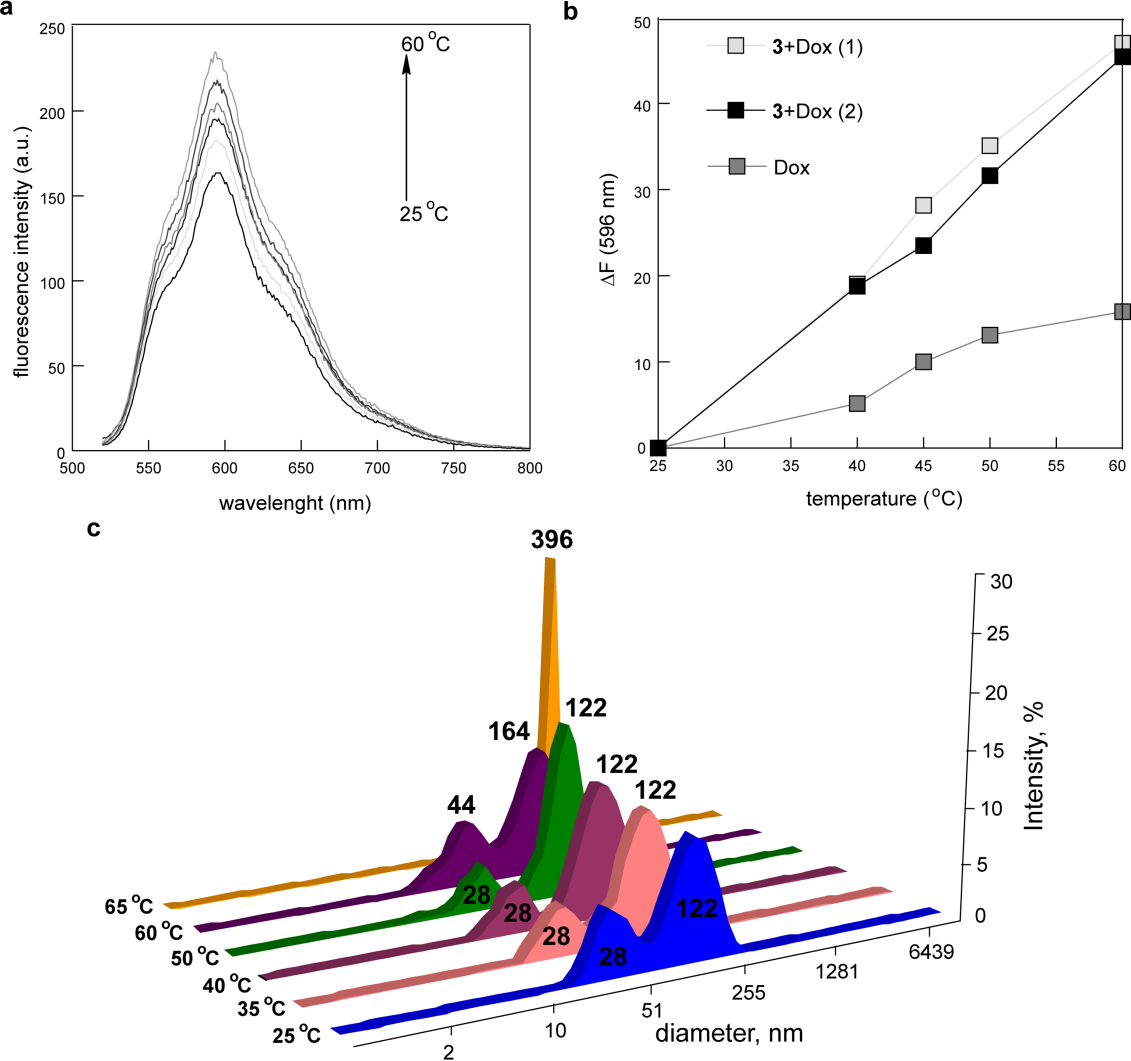
(a) Fluorescence spectra of Dox in micelles of **3** in 0.9% NaCl solution at different temperatures, *c***_3_** = 3.8 mg/mL; *c*_Dox_ = 0.3 mg/mL; λ_ex_ = 500 nm, ex/em slit 5/5, voltage: 800 V; (b) Dox fluorescence intensity change at 596 nm (Δ*F*) of the pure and micellar solutions in 0.9% NaCl as a function of the temperature, *c*_Dox_ = 0.03 mg/mL, (1): *c***_3_** = 3.8 mg/mL, *c*_Dox_ = 0.3 mg/mL, (2): *c***_3_** = 30 mg/mL, *c*_Dox_ = 0.44 mg/mL; (c) intensity-averaged particle size distribution of **3** + Dox in 0.9% NaCl solution at different temperatures, *c***_3_** = 30 mg/mL, *c*_Dox_ = 0.44 mg/mL.

As shown above, micelles of **3** exhibit temperature-dependent behaviour in the presence of salts, in particular, in 0.9% NaCl solution. So, the change of fluorescence response of the substrate while changing the temperature of **3** + Dox micellar solutions in 0.9% NaCl was studied. The binding of Dox by macrocycle **3** leads to the quenching of the substrate fluorescence *F* (Figure S11, Supporting Information File 1). Hence, the release of the substrate from **3** + Dox micelles should be accompanied by an increase of the value of *F*. The heating of **3** + Dox solutions in 0.9% NaCl to 40 °С leads to a growth of the *F* value by 18–19%, and the heating to 60 °С leads to a growth by 47–48 % (Figure 8a,b, Figure S14, Supporting Information File 1). The heating of the pure Dox solution to 40 and 60 °С increases the value of *F* by 5 and 16%, respectively (Figure 8b, Figure S14, Supporting Information File 1). This means that the *F* value mainly grows due to the release of Dox from **3** + Dox micelles, which is observed already at physiological temperature (about 40 °С).

DLS method showed a bimodal particle distribution with averaged diameters of 28 and 122 nm in **3** + Dox micelles in 0.9% NaCl solution. With increasing temperature, there is a decrease of scattering intensity of smaller particles and an increase of scattering intensity of bigger particles. At 60 °С, the particle diameters increase to 44 and 164 nm, and then the aggregation of particles into associates with an averaged diameter of 396 nm is observed (Figure 8c). It can be assumed that the amplification of hydrophobicity of micelles of **3** in the salt solution during heating leads to a weakening of their interaction with bound Dox, which in turn leads to both the increase of Dox fluorescence intensity and the growing of the size of “multimicelle aggregates” of **3**.

## Conclusion

Amphiphilic tetraundecylcalixresorcinarene **3**, bearing metoxypolyethylene glycole chains on the upper rim, forms small micellar self-organised structures with averaged diameters of 9–18 nm in aqueous solution. These structures form “multimicelle aggregates” (with average diameters of 142–164 nm) in physiological sodium chloride solution or in phosphate-buffered saline solution and become temperature-responsive. The obtained conjugate of calixresorcinarene and mPEG exhibits low hemotoxicity. It is capable to encapsulate organic compounds of various hydrophobicity including drugs. The encapsulation occurs through the penetration of substrate molecules into the macrocycle micelles and the interaction with the aromatic platform of the macrocycle. The in vitro release of substrate molecules from the micelles is slowed down (as shown by the example of an optic probe). A temperature-dependent release of substrates from **3** + Dox micelles at physiological temperature is demonstrated in 0.9% NaCl solution.

## Experimental

### Materials

Calixresorcinarene **1** was synthesized according to a previously reported procedure [36]. mPEG-550 was purchased from Aldrich, pyrene, Orange OT, ibuprofen, naproxen sodium, naproxen were purchased from Sigma-Aldrich, rhodamine B was purchased from Acros. Quercetin and doxorubicin hydrochloride were kindly supplied by Prof. E. Kh. Kazakova. Doxorubicin hydrochloride was hydrophobized by the method reported in [37]. Tosylated mPEG-550 **2** was synthesized according to a previously reported procedure [38].

### Synthesis of tetraundecylcalixresorcinarene–mPEG conjugate **3**

1 g of tetraundecylcalixresorcinarene **1** (7.2 mmol of OH group) in 100 mL of dry DMFA and 1.34 g of K_2_CO_3_ (9.72 mmol) were mixed at room temperature for 30 min under argon atmosphere. Then 8 g of **2** in 50 mL of dry DMFA was added and the reaction was carried out at 90 °С for 70 h under argon atmosphere. The solvent was evaporated under reduced pressure and the mixture was poured into a large amount of acetone to remove the inorganic salt. Then the evaporated mixture was purified through silicon-column TLC. A mixture of solvents CH_2_Cl_2_/CH_3_OH = 10:1→0:1 (v/v) was used as eluent. Pure **3** was finally recovered by dialysis (molecular weight cutoff 2000 Da) followed by evaporation of the aqueous solution and drying under reduced pressure to obtain 3.13 g (64 %) of **3**.

^1^H NMR (500 MHz, CD_3_OD) δ_H_ 0.91 (s, 12H, CH_3_), 1.30 (s, 72H, ((CH_2_)_9_), 3.37 (s, 24H, OCH_3_), 3.65 (m, 348H, OCH_2_CH_2_), 4.50 (br s, 4H, CH), 6.35 (br s, 4H, ArH^up^), 7.25 (4H, ArH^down^);

^13^C NMR (126 MHz, D_2_O) δ_C_ 13.9 CH_3_; 20.6, 22.6 (CH_2_)_9_; 29.4, 29.8 CH_2_; 32.0 ArCHAr; 58.1, 60.5 OCH_3_; 69.6, 71.1, 71.8 (CH_2_CH_2_O)*_n_*; 103, 124, 152 Ar;

IR (KBr): 3443 (ν OH, H_2_O), 2921 (ν_as_ CH_2_), 2872 (ν_s_ CH_2_), 1620 (ν C=C), 1457, 1352, 1300, 1251, 1110 (ν_as_ C-O-C); 953, 850 (ν C-C-O) cm^−1^.

### Methods

^1^H and ^13^C NMR spectra were measured on a Bruker AVANCE(III) 500 spectrometer (500 MHz for ^1^H NMR, 126 MHz for ^13^C NMR). Chemical shifts were reported relative to residual solvent protons as an internal standard.

MALDI mass spectra were recorded on an Ultra Flex III TOF/TOF mass spectrometer (Bruker Daltonic, Bremen, Germany) operated in the linear mode. A Nd:YAG laser, λ = 355 nm was used. The data were processed using the FlexAnalysis 3.0 software (Bruker Daltonics, Bremen, Germany). Positively charged ions were registered. A metal target MTP AnchorChip^TM^ was used. 2,5-Dihydroxybenzoic acid (DHB) of chemical grade was used as a matrix. Portions (0.5 μL) of a 1% matrix solution in acetonitrile and of a 0.1% sample solution in methanol were consecutively applied onto the target and evaporated. An aqueous solution of cesium chloride (CsCl) at a concentration of 1 mg/mL was added to improve the ionization of the analyzed sample.

IR spectra were recorded with a Bruker Vector 22 FTIR Spectrometer (Bruker Optics, USA) in KBr pellets. Fluorescence spectra were recorded on a Varian CaryEclipse spectrofluorimeter equipped with a Cary Temperature Controller. Absorbance measurements were performed in a Lambda 35 UV–vis spectrometer (Perkin Elmer Instruments) in quartz cells. Absorbance measurements at different temperatures were carried out using a Specord 50 plus instrument of Analytic Jena AG in quartz cells of 1 cm path length.

The hemolytic activity of C11-mPEG against human red blood cells (hRBC) was tested by the method reported in [39].

Small-angle X-ray scattering (SAXS) data for samples were collected with the Bruker AXS Nanostar SAXS system using Cu Kα (λ = 1.5418 Å) radiation from a 2.2 kW X-ray tube (40 kV, 35 mA) coupled with Göbel mirror optics and a HiStar 2D area detector. The beam was collimated using three pinholes with apertures of 800, 450 and 700 μm. The instrument was operated with a sample-to-detector distance of 63.5 cm to provide data at angles of 0.1° < 2θ < 4.8°, which corresponds to 0.007 Å^−1^ < *s* < 0.34 Å^−1^. The value of *s* is proportional to the inverse of the length scale (*s* = (4π/λ)sin(θ) in units of Å^−1^). Scattering patterns were obtained for the samples at 23 °C in an evacuated chamber. The measurements were performed in transition mode with the use of glass capillaries filled by liquid sample and water. The capillaries (2 mm diameter) were sealed and put into the evacuated chamber by means of the holders. For each sample several experiments were performed. The results of the experiments are summarized, so that the total time of each experiment was equal to 30000 sec. The 2D scattering patterns were integrated using the SAXS program package [40]. Calculation of structural parameters, simulation, and graphical representation of the results were performed using PRIMUS [26] and SASView [41] software packages.

DLS, SLS, and zeta-potential measurements were carried out by employing a Zetasizer nano ZS with Dispersion Technology Software 5.00. The measurements were carried out at 25 °C in polystyrene cells, for temperature-dependent DLS measurements (25–65 °C) and SLS measurement, a glass cuvette PCS8501 (Malvern) was utilized. The SLS measurements were carried out in ethanol solution. Zeta-potential measurements were carried out in folded capillary cells DTS1061 (Malvern). Zeta-potential values were calculated from electrophoretic mobilities by using the Hückel approximation for solutions of low ionic strength.

TEM images were obtained in a Libra 120 (Carl Zeiss) microscope. The images were acquired at an accelerating voltage of 100 kV. Samples were dispersed on 300 mesh copper grids with continuous carbon-formvar support films.

#### Determination of cac values

The critical association concentration (cac) of **3** in aqueous solution was determined by fluorescence spectra of pyrene (0.002 mM). The concentration of **3** and mPEG-550 was varied from 0.001 to 0.9 mg/mL. Pyrene was excited at 333 nm, emission spectra were recorded in the range of 338–500 nm, the excitation and emission slit widths were 2.5 nm, 1 cm quartz cuvettes were employed at 25 °C. The ratio of first (372 nm) and third (381 nm) emission band for every spectrum was estimated and the cac values were determined from the dependence of I/III on the concentration of macrocycle or mPEG-550.

#### Preparation of micelles of **3** for DLS and TEM

The thin-film hydration method was used to prepare micelles of **3**. The compound (160 mg) was dissolved in 1 mL of dehydrated ethanol, the solvent was evaporated in a rotary evaporator at 25 °C to obtain a thin film. The obtained film was hydrated in 1 mL water under moderate shaking at 25 °C and filtrated through Millipore ﬁlter (pore size: 0.2 μm). The series of solutions was obtained through dilution.

#### Preparation of substrate-loaded micelles of **3**

Two basic methods were used to produce **3** + substrate micelles.

#### Thin-film hydration

Appropriate amounts of **3** and substrate (Dox, Orange OT, QC, RhB, see Table S1, Supporting Information File 1) were dissolved in 1 mL of dehydrated ethanol and mixed for 30 min at 25 °C. Then the solvent was evaporated using a rotary evaporator at 25 °C to obtain a thin film. The obtained films were hydrated in 1 mL water under moderate shaking at 25 °C. Finally, the residual suspensions were ﬁltered through Millipore ﬁlter (pore size: 0.2 μm) to remove free hydrophobic substrates (Dox, Orange OT, QC) or dialysed against water (molecular weight cutoff 1000 Da) for 1 h (hydrophilic RhB). The concentrations of Dox, Orange OT, QC, RhB in the obtained **3** + substrate micelles were determined by through absorption measurements in water/ethanol 1:1 solutions of micelles to avoid the influence of macrocycle–substrate complexation on the absorbance intensity of substrate. QC was quantiﬁed by measuring the absorbance at 374 nm using ε = 28400 М^−1^·cm^−1^ [42], Оrange OT at 490 nm using ε = 18600 М^−1^·cm^−1^ [43], Dox at 553 nm using ε = 15357 М^−1^·cm^−1^ [44], RhB in aqueous solution at 554 nm using ε = 10800 М^−1^·cm^−1^ [45].

#### Solubilisation

To two solutions of **3** (10 mg) in D_2_O (1 mL) 10 mg of naproxen or ibuprofen were added and the solutions were mixed for 5 h at 25 °C at a rate of 360 rpm. Then they were centrifuged (6000 rpm, 10 min, centrifuge Eva-20 (Hettich Zentrifugen, Germany)), non-solubilized substrate was filtered and washed with ice water (5 mL), dried under reduced pressure and weighed. The amount of solubilized substrate was determined by subtraction of the mass of non-solubilised substrate from the total substrate mass.

The drug loading (DL) and encapsulation efﬁciency (EE) values were calculated using the following equations:

[1]
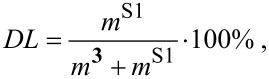


[2]
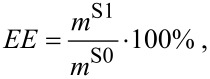


where *m***^3^** is the mass of **3** (mg), *m*^S0^ is the initial mass of substrate (mg) and *m*^S1^ is the mass of solubilized substrate (mg).

#### Release of RhB from **3** + RhB micelles in vitro

**3** + RhB micelles solution (2 mL) was transferred into a dialysis bag (molecular weight cutoff 1000 Da) and placed in 200 mL of phosphate-buffered saline solution (pH 7.4). The release study was performed at 37 °C in a thermostatic bath at a mixing rate of 300 rpm. 3 mL of the buffered solution outside the dialysis bag were removed for analysis at suitable intervals, and replaced with fresh buffer solution. RhB concentration was determined by measuring the absorption at 554 nm using ε = 10800 М^−1^·cm^−1^ [45].

## Supporting Information

File 1 Additional experimental data.
