# Supplementary material for: Nanoconjugates of a calixresorcinarene derivative with methoxy poly(ethylene glycol) fragments for drug encapsulation
Source: Beilstein J Nanotechnol. 2018 Jul 27;9:2057–70. doi: 10.3762/bjnano.9.195 (PMC6071701; doi:10.3762/bjnano.9.195)
Supplement: File 1 — Additional experimental data. [file Beilstein_J_Nanotechnol-09-2057-s001.pdf]

Supporting Information

for

Nanoconjugates of a calixresorcinarene derivative with methoxy poly(ethylene glycol) fragments for drug encapsulation

Alina M. Ermakova[1,2], Julia E. Morozova*[1,2], Yana V. Shalaeva[1,2], Victor V. Syakaev[1], Aidar T. Gubaidullin[1], Alexandra D. Voloshina[1], Vladimir V. Zobov[1], Irek R. Nizameev[1,3], Olga B. Bazanova[1], Igor S. Antipin[1,2] and Alexander I. Konovalov[1]

Address: [1]Arbuzov Institute of Organic and Physical Chemistry, FRC Kazan Scientific Center of RAS, Arbuzov str. 8, 420088 Kazan, Russian Federation; [2]Kazan Federal University, Kremlevskaya st. 18, 420008 Kazan, Russian Federation and [3]Kazan National Research Technical University named after A. N. Tupolev – KAI, K. Marx str. 10, 420111 Kazan, Russian Federation

Email: Julia E. Morozova* - ernestina34@gmail.com

* Corresponding author

Additional experimental data

**Contents**	**Page**

Figure S1: ^{13}C NMR spectra of macrocycle **1** in acetone-d_6 (top) and of macrocycle **3** in D_2O (bottom). S4

Figure S2: ^{1}H NMR spectrum of **3** in CD_3OD. S4

Figure S3: Debye plots for **3** in ethanol solution, c = 1–160 mg/mL. S5

Figure S4: MALDI-TOF mass spectrum of **3** (DHB, in the presence of CsCl). S5

Figure S5: SAXS diffraction intensity profiles at 23 °C (logarithmic scale) of an aqueous solution of **3** (c = 137 mg/mL, blue curve) and water (red curve) in 2 mm glass capillaries. Scattering vector $s = 4\pi \sin\theta/\lambda$, $Å^{-1}$; λ = 1.5418 Å is the X-ray wavelength. S6

Figure S6: (a) The fitting of experimental SAXS curve and (b) calculated distance distribution functions $p(r)$ for the sample; circles: measurement, solid line: calculated curves. S6

Figure S7: MALDI-TOF mass spectrum of mPEG-550 (*p*-NA). S7

Figure S8: The dependence of pyrene I/III in aqueous solutions of macrocycle **3** and the pure mPEG-550. S7

Table S1: The DLS data for aqueous solutions of **3**. S8

Figure S9: Zeta potential of **3** particles in aqueous solution (c = 5 mg/mL), 25 °C. S9

Figure S10: Intensity-averaged particle size distribution for a solution of **3** in PBS (pH 7.4) at different temperatures ($c_{\mathbf{3}}$ = 10 mg/mL). S9

Figure S11: (a) Intensity-averaged particle size distribution for a solution of **3** + Dox, $c_{\mathbf{3}}$ = 30 mg/mL; (b) fluorescence spectra of Dox in PBS solution (c_{Dox} = 0.03 mg/mL) and of **3** + Dox micelles in PBS solution; (1): $c_{\mathbf{3}}$ = 3.8 mg/mL, c_{Dox} = 0.3 mg/mL; (2): $c_{\mathbf{3}}$ = 30 mg/mL, c_{Dox} = 0.4 mg/mL; λ_{ex} = 500 nm, ex/em slit 5/5, voltage: 800 V; (c, d) fluorescence spectra of (c) Dox·HCl in PBS solution and of mPEG-550+Dox·HCl in PBS solution and of (d) **3** + Dox·HCl in PBS solution; c_{Dox} = 0.14 mg/mL; $c_{\mathbf{3}}$ = 3.3 mg/mL, c_{mPEG-} S10

$_{550}$ = 9.9 mg/mL; λ_{ex} = 500 nm; (c) ex/em slit 2.5/2.5, voltage: 700 V, (d) ex/em slit 5/2.5, voltage: 800 V. The spectra of **3** in PBS solution, the spectra of mPEG-550 in PBS solution, and the spectra of PBS are added as reference.

Figure S12: Fluorescence spectra of Naproxene sodium (NS) in PBS solution, and in the presence of mPEG-550 and **3** in PBS solution. The spectra of **3** in PBS solution and the spectra of PBS are added as a reference. c_{NS} = 1 mM, $c_{\mathbf{3}}$ = 6.7 mg/mL; c_{mPEG} = 5.8 mg/mL; λ_{ex} = 279 nm, ex/em slit 1.5/2.5, voltage: 800 V. S11

Figure S13: (a) Fluorescence spectra of RhB in PBS solution, and in the presence of mPEG-550 and **3** in PBS solution. The spectrum of PBS solution is added as a reference. c_{RhB} = 0.01 mM, $c_{\mathbf{3}}$ = 19 mg/mL, C_{mPEG} = 15.4 mg/mL; λ_{ex} = 554 nm, ex/em slit 1.5/1.5, voltage: 800 V; (b) intensity-averaged particle size distribution for solutions of **3** ($c_{\mathbf{3}}$ = 160 mg/mL) and **3** + RhB ($C_{\mathbf{3}}$ = 150 mg/mL). S11

Figure S14: Fluorescence spectra of Dox in 0.9% NaCl solution at different temperatures. (a) Pure Dox solution, c_{Dox} = 0.03 mg/mL; (b) **3** + Dox micellar solution, $c_{\mathbf{3}}$ = 30 mg/mL, C_{Dox} = 0.44 mg/mL; λ_{ex} = 500 nm, ex/em slit 5/5, voltage: 800 V. S12

References S12

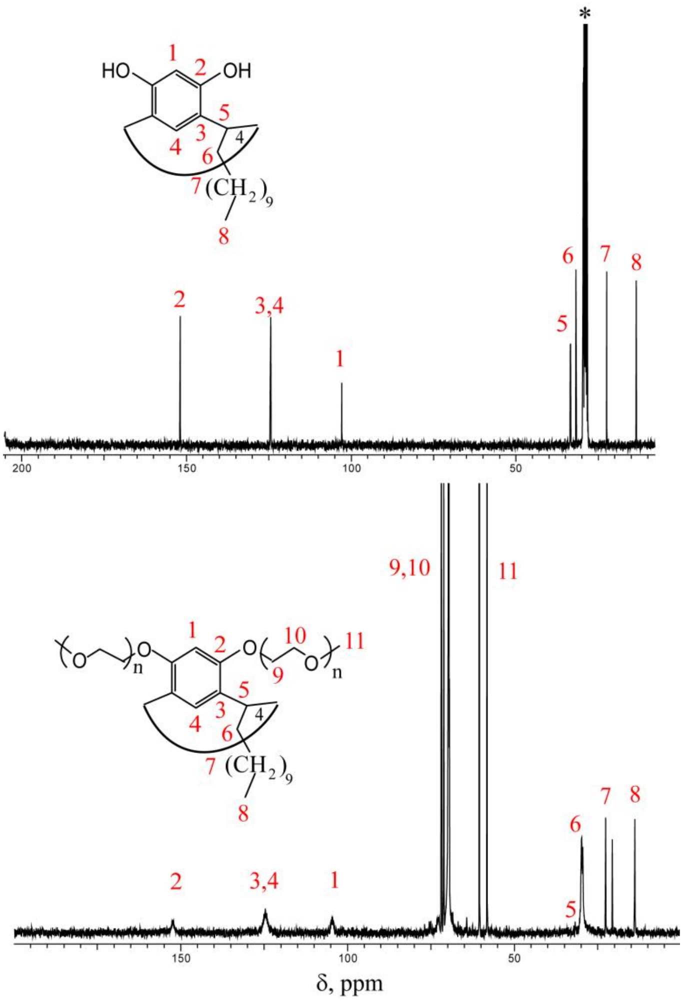

Figure S1: ^{13}C NMR spectra of macrocycle **1** in acetone-d_6 (top) and of macrocycle **3** in D_2O (bottom).

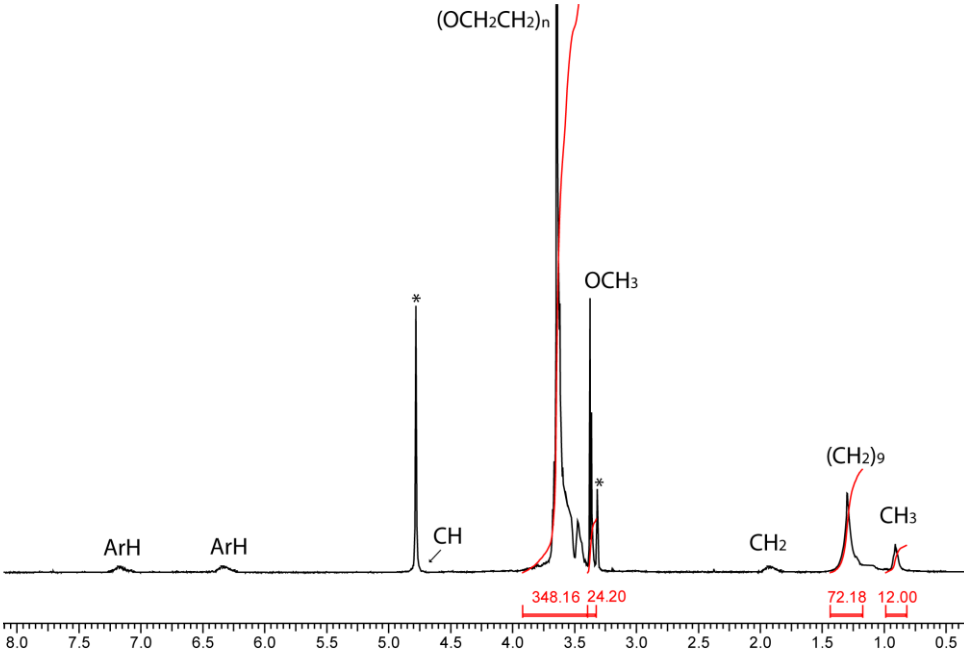

Figure S2: ^{1}H NMR spectrum of **3** in CD_3OD.

In the ^{1}H NMR spectrum of **3**, the ratios between integral intensities of terminal methoxy groups (at 3.37 ppm) and methyl (at 0.91 ppm) and $(CH_2)_9$ groups (at 1.30 ppm) are 6/3 and 6/18, respectively. This testifies the complete substitution of the hydroxyl groups of the macrocycle **1** and allows one to estimate the common quantity of attached ethyleneoxygroups and the average molecular mass of **3**. The integral intensity of the signal at 3.65 ppm corresponds to approximately 86 ethyleneoxy groups per one macrocycle molecule, which gives an average molecular mass about 5.0 kDa.

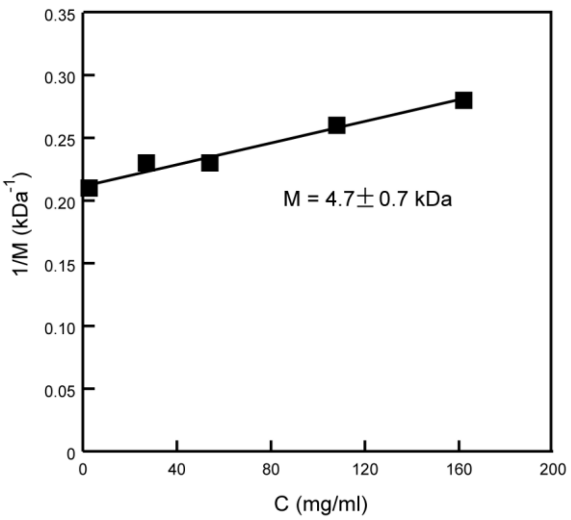

Figure S3: Debye plots for **3** in ethanol solution, c = 1–160 mg/mL.

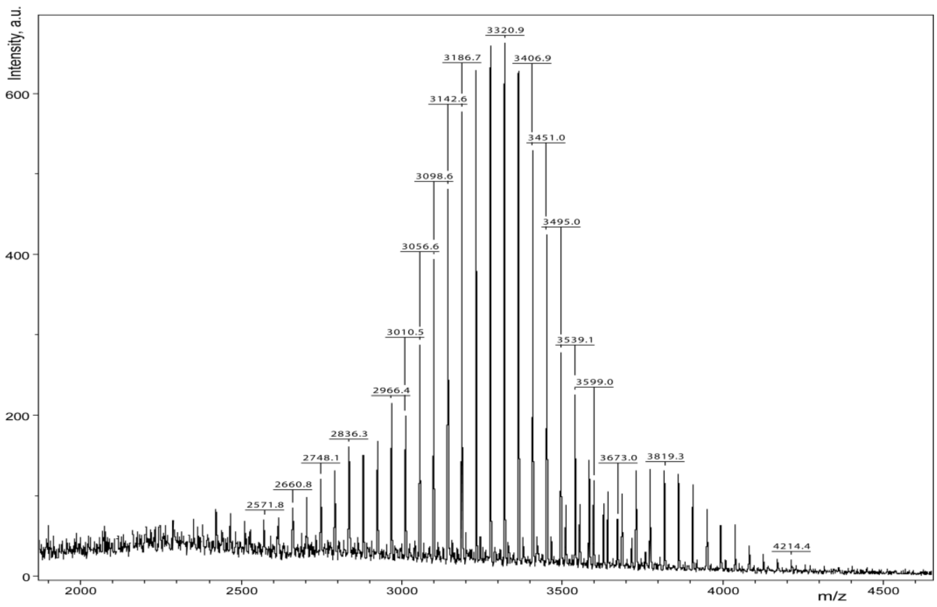

Figure S4: MALDI-TOF mass spectrum of **3** (DHB, in the presence of CsCl).

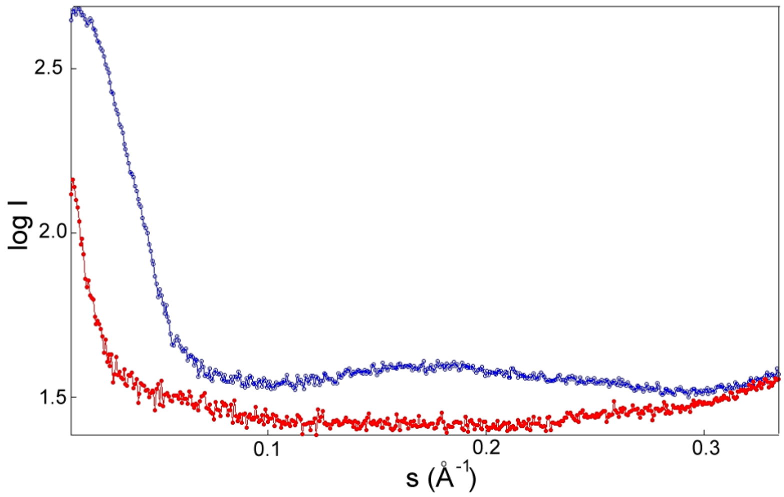

Figure S5: SAXS diffraction intensity profiles at 23 °C (logarithmic scale) of an aqueous solution of **3** (c = 137 mg/mL, blue curve) and water (red curve) in 2 mm glass capillaries. Scattering vector $s = 4\pi \sin\theta/\lambda$, Å^{-1}; λ = 1.5418 Å is the X-ray wavelength.

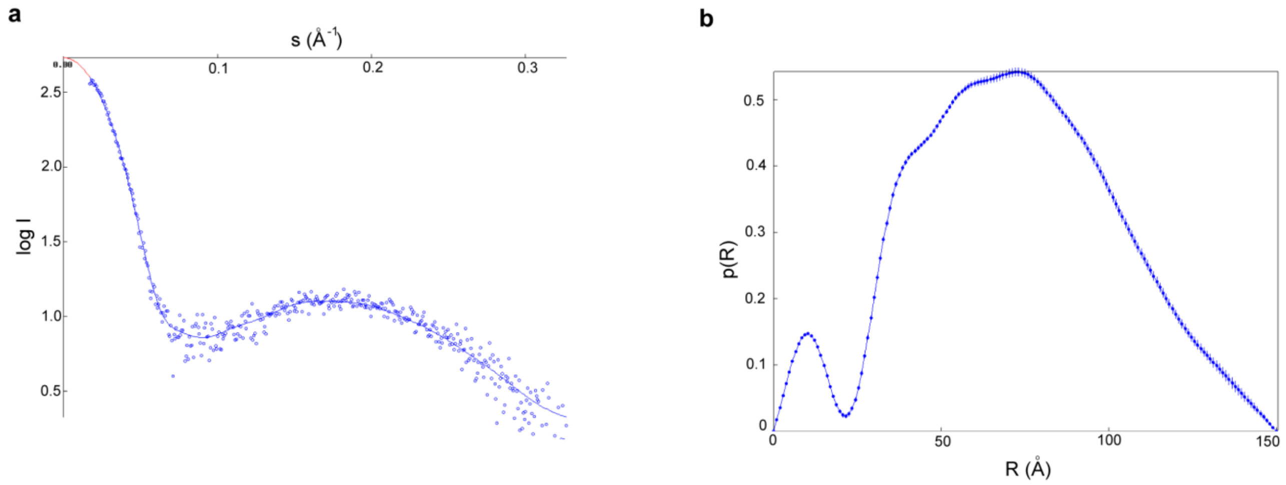

Figure S6: (a) The fitting of experimental SAXS curve and (b) calculated distance distribution functions $p(r)$ for the sample; circles: measurement, solid line: calculated curves.

The values of radius of gyration R_g (53.5 ± 1.8 Å, by Guinier plot) in the case of a sphere-shape model correspond to an average effective particles radius of 71.5 Å (diameter of about 143 Å). Taking into account the values of the average lengths of hydrophobic tail and phenyl ring of macrocycle (about 13.9 Å and 2.8 Å, respectively), the average thickness of hydrophilic shell, consisting of mPEG chains, is about 54.8 Å. The effective length of an oxyethylene group is about 3.5 Å [1]), therefore the maximum number of the oxyethylene groups in the mPEG chains attached to OH groups of macrocycle platform is 15–16. This is consistent with the distribution of oxyethylene groups in the initial mPEG-550 (see below).

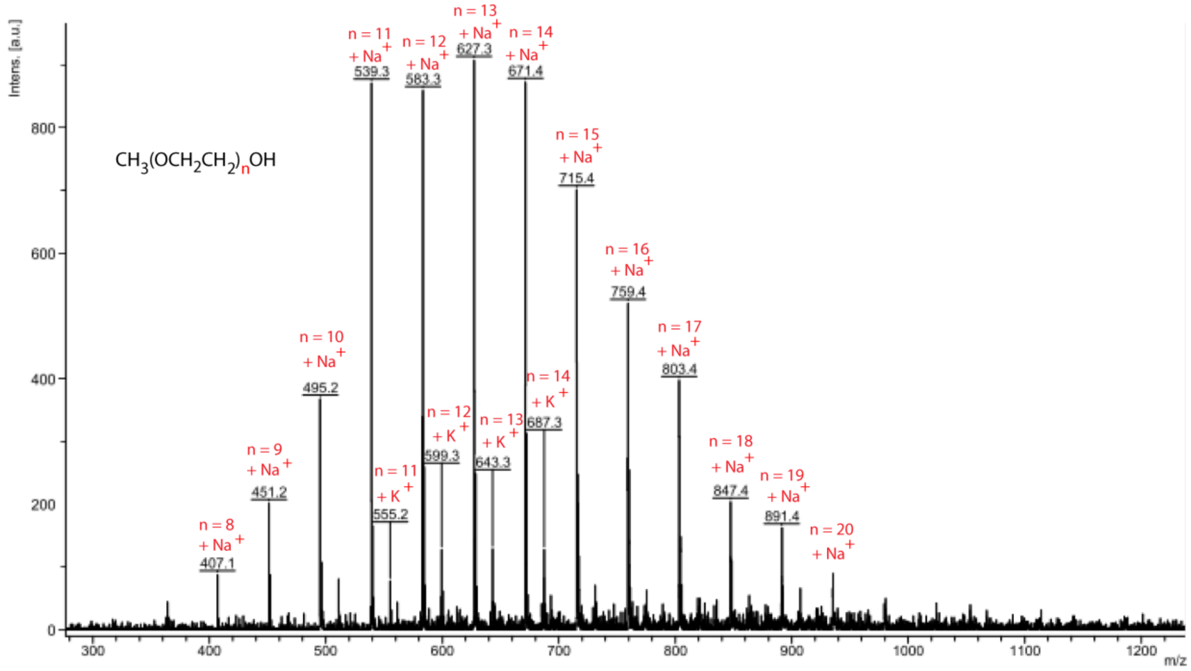

Figure S7: MALDI-TOF mass spectrum of mPEG-550 (*p*-NA).

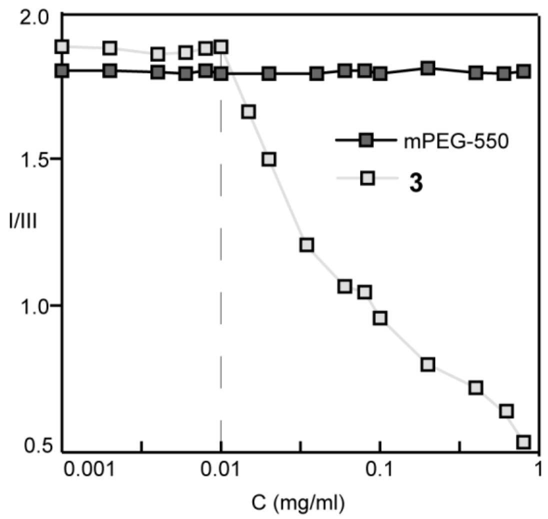

Figure S8: The dependence of pyrene I/III in aqueous solutions of macrocycle **3** and the pure mPEG-550.

Table S1: The DLS data for aqueous solutions of **3**.

c_3, mg/mL	addition	*T*, °C	*I* (%)[a]	*N* (%)[b]	*V* (%)[c]	PDI
160	—	25	18.17 (11); 531.2 (3.6)	10.1 (17.2)	11.7 (16.3)	0.518
100	—	25	11.7 (13); 68.06 (1.8); 396.1 (3.2)	7.531 (21)	8.721 (19.6)	0.465
55	—	25	8.721 (13.1); 122.4 (3.3)	5.615 (15.1)	6.503 (16.4)	0.366
30	—	25	8.721 (12.8); 164.2 (3.1)	7.531 (33.3)	7.531 (30.6)	0.624
10	—	25	8.721 (17.3); 58.77 (4.9)	6.503 (29.5)	7.531 (26.8)	0.365
3	—	25	8.721 (12.8); 58.77 (11.7)	7.531 (33.3)	7.531 (30.6)	0.624
experiments at different temperatures						
10	—	25	8.721 (16.4); 68.06 (6.1)			0.394
10	—	35	8.721 (14.9); 78.8 (4.8)			0.357
10	—	45	10.1 (23.4); 105.7 (5.5)			0.311
10	—	55	10.1 (16.9); 91.28 (4.5)			0.294
10	—	60	10.1 (17.6); 91.28 (4.7)			0.280
10	—	65	11.7 (20.7); 105.7 (4.7)			0.268
10	0.9 % NaCl	25	164.2 (27.9)			0.232
10	0.9 % NaCl	35	190.1 (14.8)			0.275
10	0.9 % NaCl	45	190.1 (17.8)			0.273
10	0.9 % NaCl	55	220.2 (12.5)			0.340
10	0.9 % NaCl	60	342 (25)			0.154
10	0.9 % NaCl	65	396 (33)			0.132
10	PBS	25	15.69 (8.1); 141.8 (17.2)			0.600
10	PBS	35	18.17 (4.7); 164.2 (9.2)			0.577
10	PBS	45	18.17 (4.7); 164.2 (12.1)			0.582
10	PBS	55	21.04 (8.4); 164.2 (15.1)			0.522
10	PBS	60	28.21 (7.2); 220.2 (12.5)			0.273
10	PBS	65	28.21 (9); 396 (19)			0.275
Encapsulation of substrates						
10	QC	25	10.1 (12.4); 105.7 (8.2)	8.721 (12.2)	8.721 (11.6)	0.566
10	Orange OT	25	10.1 (26.9); 342 (15.3)	8.721 (35.3)	10.1 (36.6)	0.602
30	Dox	25	15.69 (16.4)	11.7 (26.9)	11.7 (22.4)	0.205
150	RhB	25	28.21 (8.5); 255 (4.7)	8.721 (25.1)	8.721 (17.2)	0.537

[a] intensity-averaged particles size distribution; [b] number-averaged particles size distribution; [c]intensity-averaged particles size distribution

			Mean (mV)	Area (%)	Width (mV)
Zeta Potential (mV):	-10.1	Peak 1:	-10.1	100.0	4.82
Zeta Deviation (mV):	4.82	Peak 2:	0.00	0.0	0.00
Conductivity (mS/cm):	0.136	Peak 3:	0.00	0.0	0.00

Result quality : Good

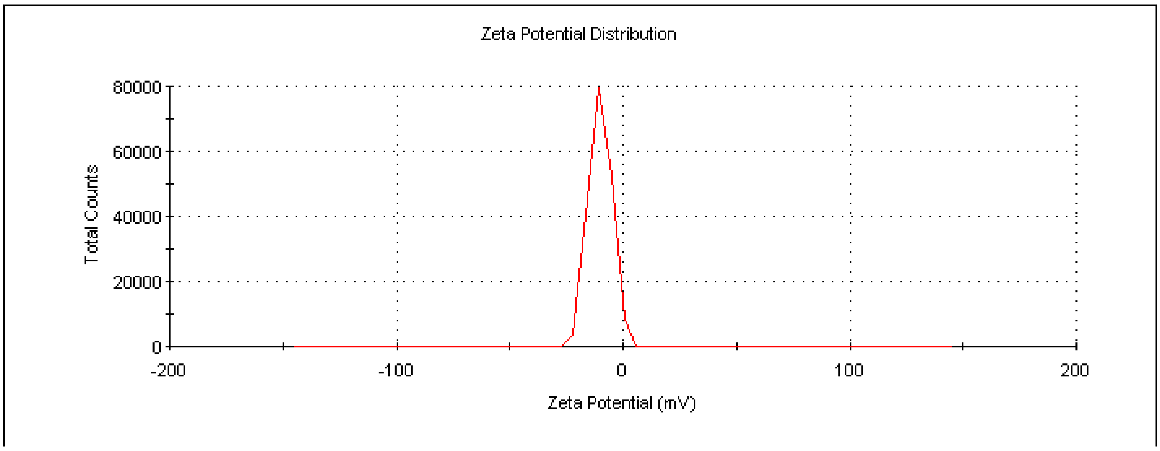

Figure S9: Zeta potential of **3** particles in aqueous solution (*c* = 5 mg/mL), 25 °C.

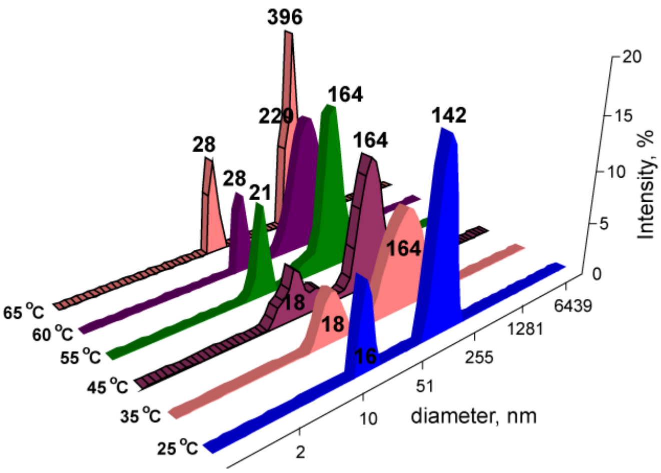

Figure S10: Intensity-averaged particle size distribution for a solution of **3** in PBS (pH 7.4) at different temperatures (c_3 = 10 mg/mL).

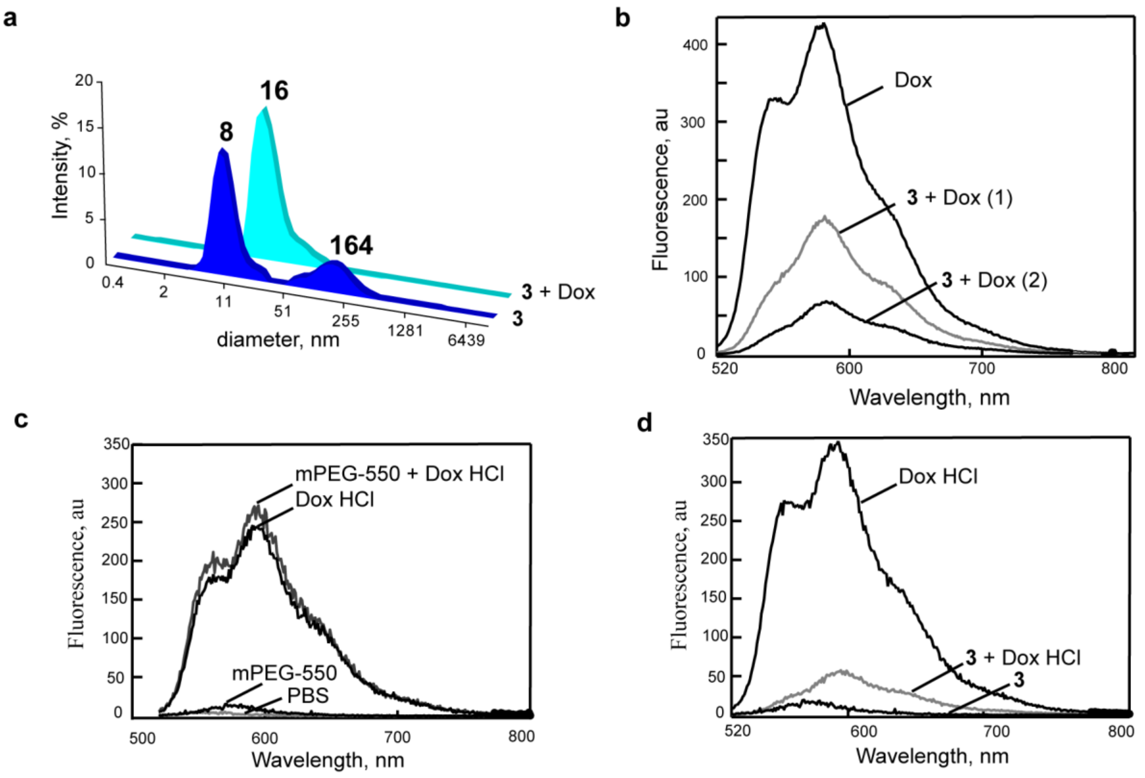

Figure S11: (a) Intensity-averaged particle size distribution for a solution of **3** + Dox, c_3 = 30 mg/mL; (b) fluorescence spectra of Dox in PBS solution (c_{Dox} = 0.03 mg/mL) and of **3** + Dox micelles in PBS solution; (1): c_3 = 3.8 mg/mL, c_{Dox} = 0.3 mg/mL; (2): c_3 = 30 mg/mL, c_{Dox} = 0.4 mg/mL; λ_{ex} = 500 nm, ex/em slit 5/5, voltage: 800 V; (c, d) fluorescence spectra of (c) Dox·HCl in PBS solution and of mPEG-550+Dox·HCl in PBS solution and of (d) **3** + Dox·HCl in PBS solution; c_{Dox} = 0.14 mg/mL; c_3 = 3.3 mg/mL, $c_{mPEG-550}$ = 9.9 mg/mL; λ_{ex} = 500 nm; (c) ex/em slit 2.5/2.5, voltage: 700 V, (d) ex/em slit 5/2.5, voltage: 800 V. The spectra of **3** in PBS solution, the spectra of mPEG-550 in PBS solution, and the spectra of PBS are added as reference.

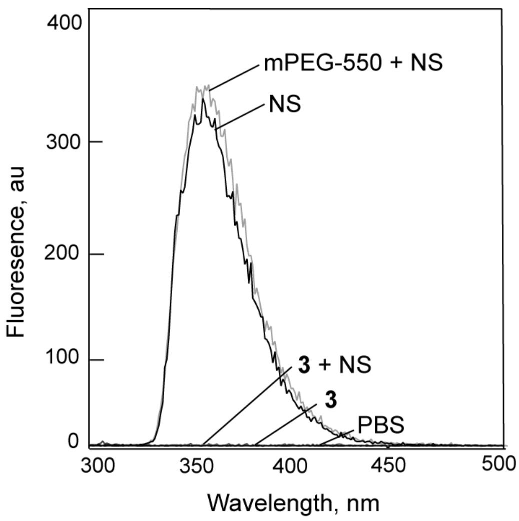

Figure S12: Fluorescence spectra of Naproxene sodium (NS) in PBS solution, and in the presence of mPEG-550 and **3** in PBS solution. The spectra of **3** in PBS solution and the spectra of PBS are added as a reference. c_{NS} = 1 mM, $c_{\mathbf{3}}$ = 6.7 mg/mL; c_{mPEG} = 5.8 mg/mL; λ_{ex} = 279 nm, ex/em slit 1.5/2.5, voltage: 800 V.

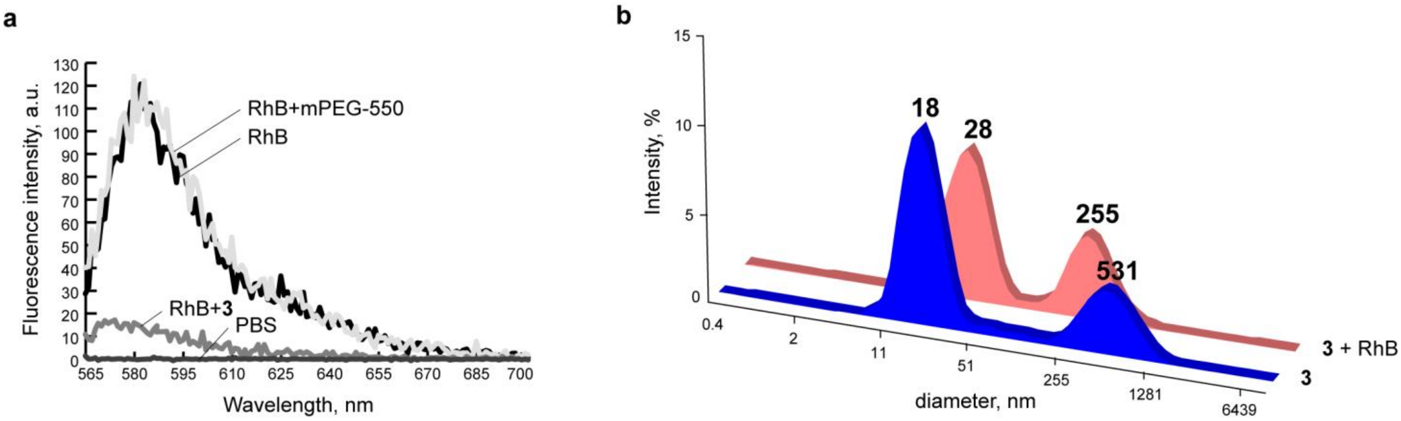

Figure S13: (a) Fluorescence spectra of RhB in PBS solution, and in the presence of mPEG-550 and **3** in PBS solution. The spectrum of PBS solution is added as a reference. c_{RhB} = 0.01 mM, $c_{\mathbf{3}}$ = 19 mg/mL, C_{mPEG} = 15.4 mg/mL; λ_{ex} = 554 nm, ex/em slit 1.5/1.5, voltage: 800 V; (b) intensity-averaged particle size distribution for solutions of **3** ($c_{\mathbf{3}}$ = 160 mg/mL) and **3** + RhB ($C_{\mathbf{3}}$ = 150 mg/mL).

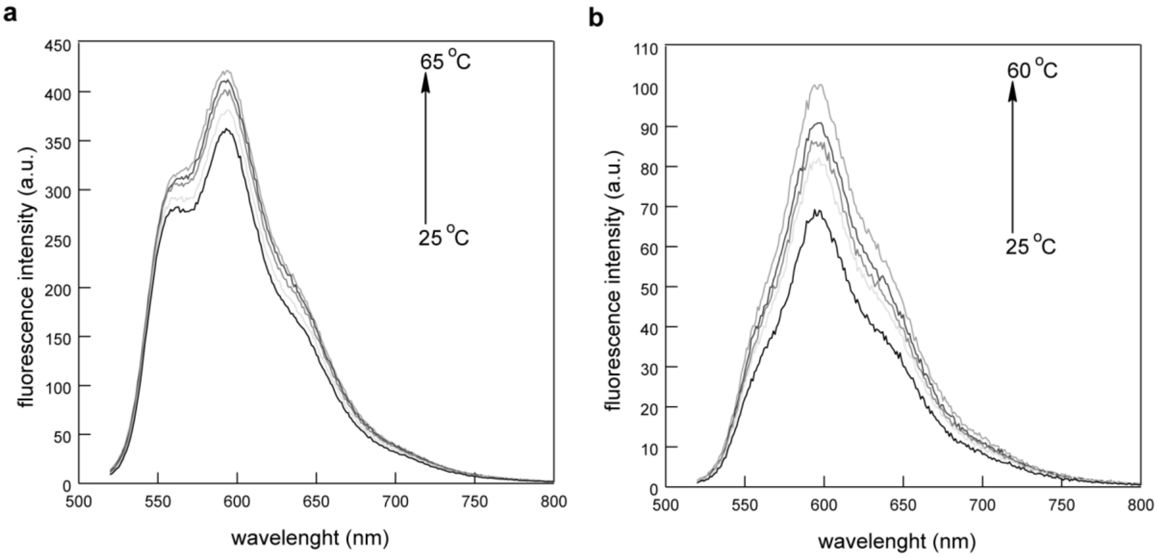

Figure S14: Fluorescence spectra of Dox in 0.9% NaCl solution at different temperatures. (a) Pure Dox solution, c_{Dox} = 0.03 mg/mL; (b) **3** + Dox micellar solution, $c_{\mathbf{3}}$ = 30 mg/mL, C_{Dox} = 0.44 mg/mL; λ_{ex} = 500 nm, ex/em slit 5/5, voltage: 800 V.

References

1. Kenworthy, A. K.; Hristova, K.; Needham, D.; McIntosh, T.J. *Biophys. J.* **1995**, *68*, 1921–1995. doi:10.1016/S0006-3495(95)80369-3.
